# Supplementary figures and images for: Mesenchymal stem cells promote metastasis through activation of an ABL-MMP9 signaling axis in lung cancer cells
Source: PLoS One. 2020 Oct 29;15(10):e0241423. doi: 10.1371/journal.pone.0241423 (PMC7595271; doi:10.1371/journal.pone.0241423)

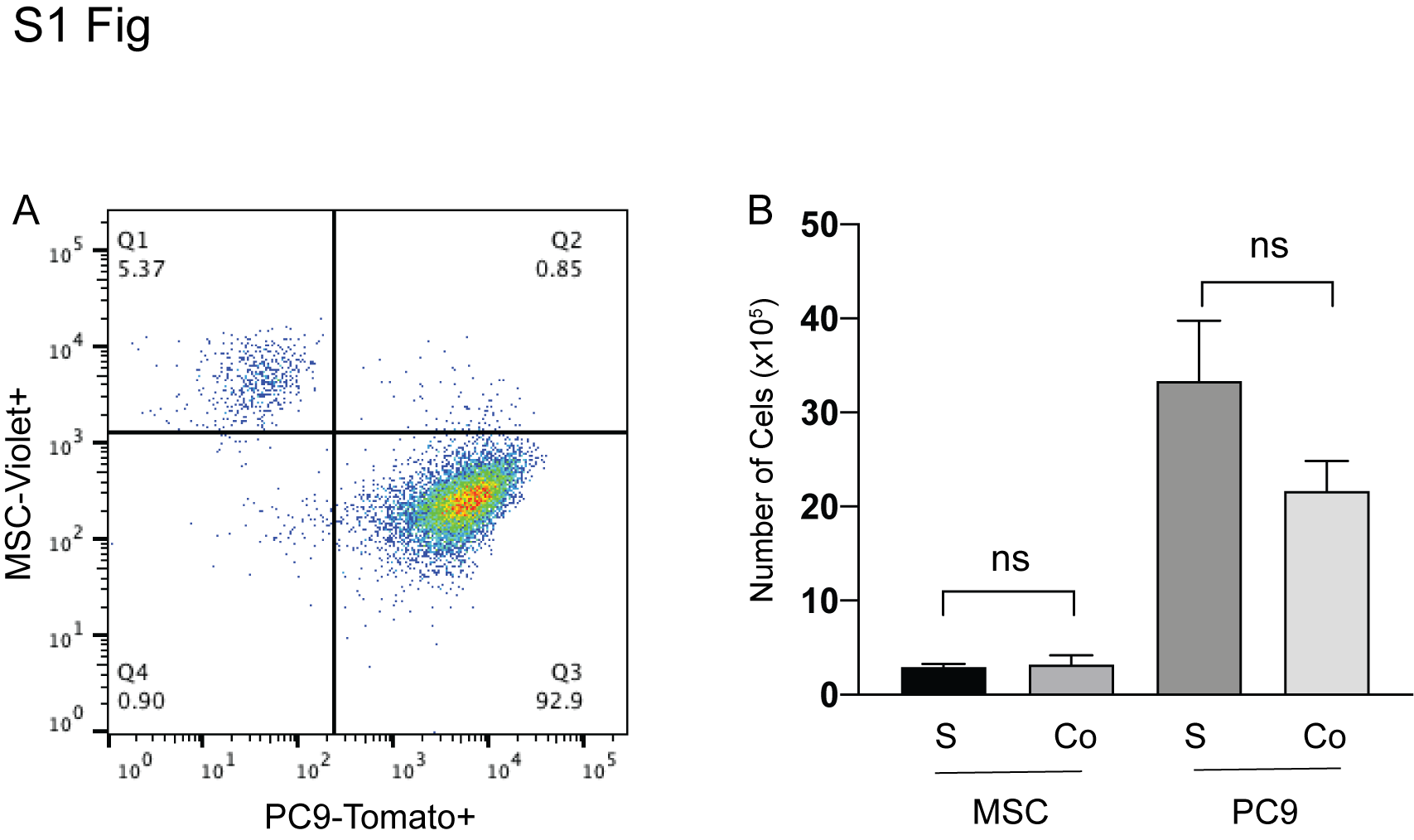

Supplement: S1 Fig — PC9 cells labeled with pFU-luciferase tomato were co-cultured with Cell-Tracker violet-labeled MSCs at 1:1 ratio. (A) Representative flow cytometry cell sorting to separate PC9 cells from MSCs after 3 days of co-culture. (B) Comparison of MSC and PC9 cell numbers grown under single culture (S) versus co-culture (Co) conditions and separated by FACS (n = 3). (TIF) [file pone.0241423.s001.tif]

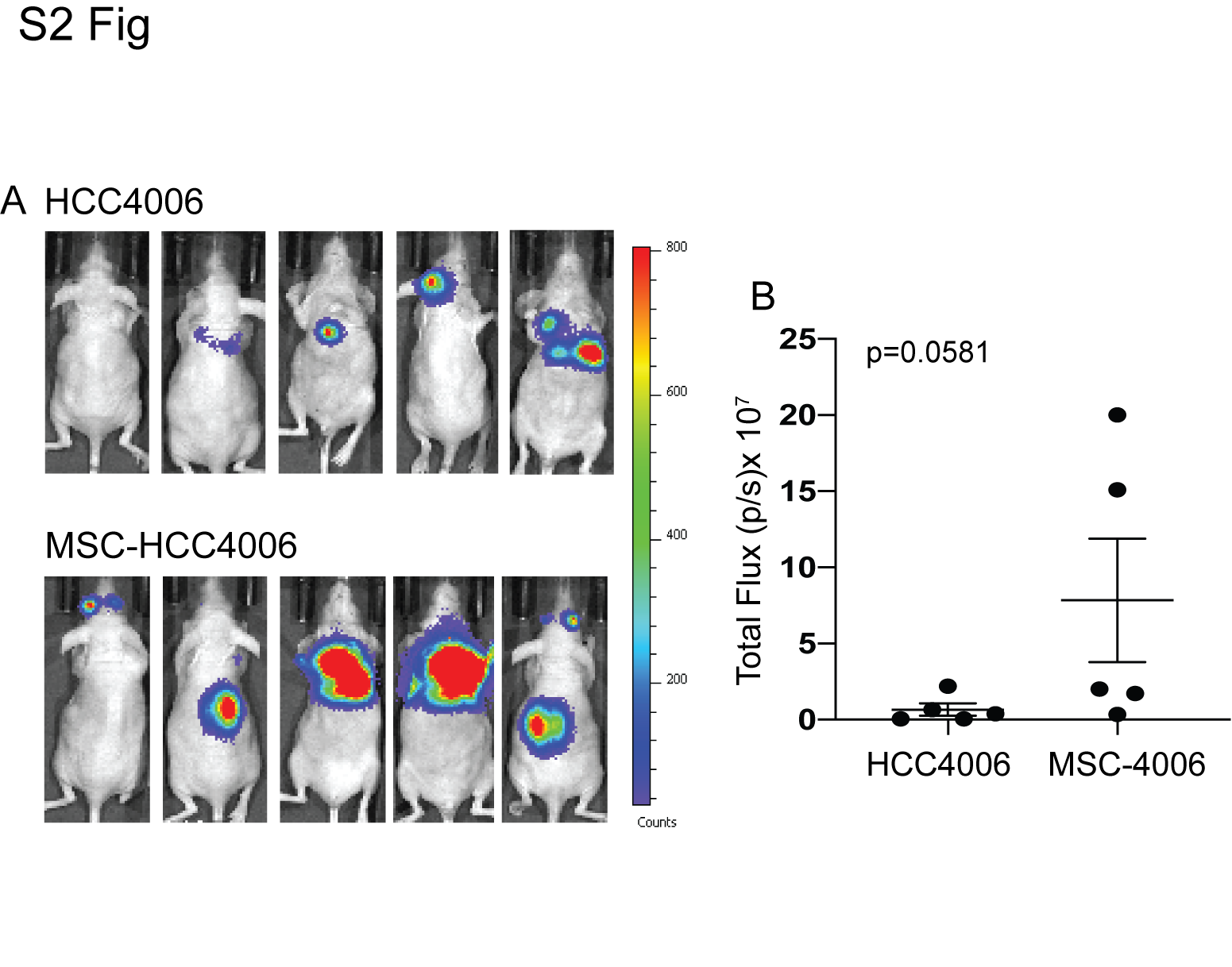

Supplement: S2 Fig — (A) HCC4006 cells labeled with luciferase were cultured with or without MSCs (1:1 ratio). Equal number of cells were used for intracardiac injection and BLI imaging was performed at day 28 post-injection. (B) Total flux (p/s) counts was plotted (n = 5). Statistical p value was calculated using unpaired one-tailed t test. (TIF) [file pone.0241423.s002.tif]

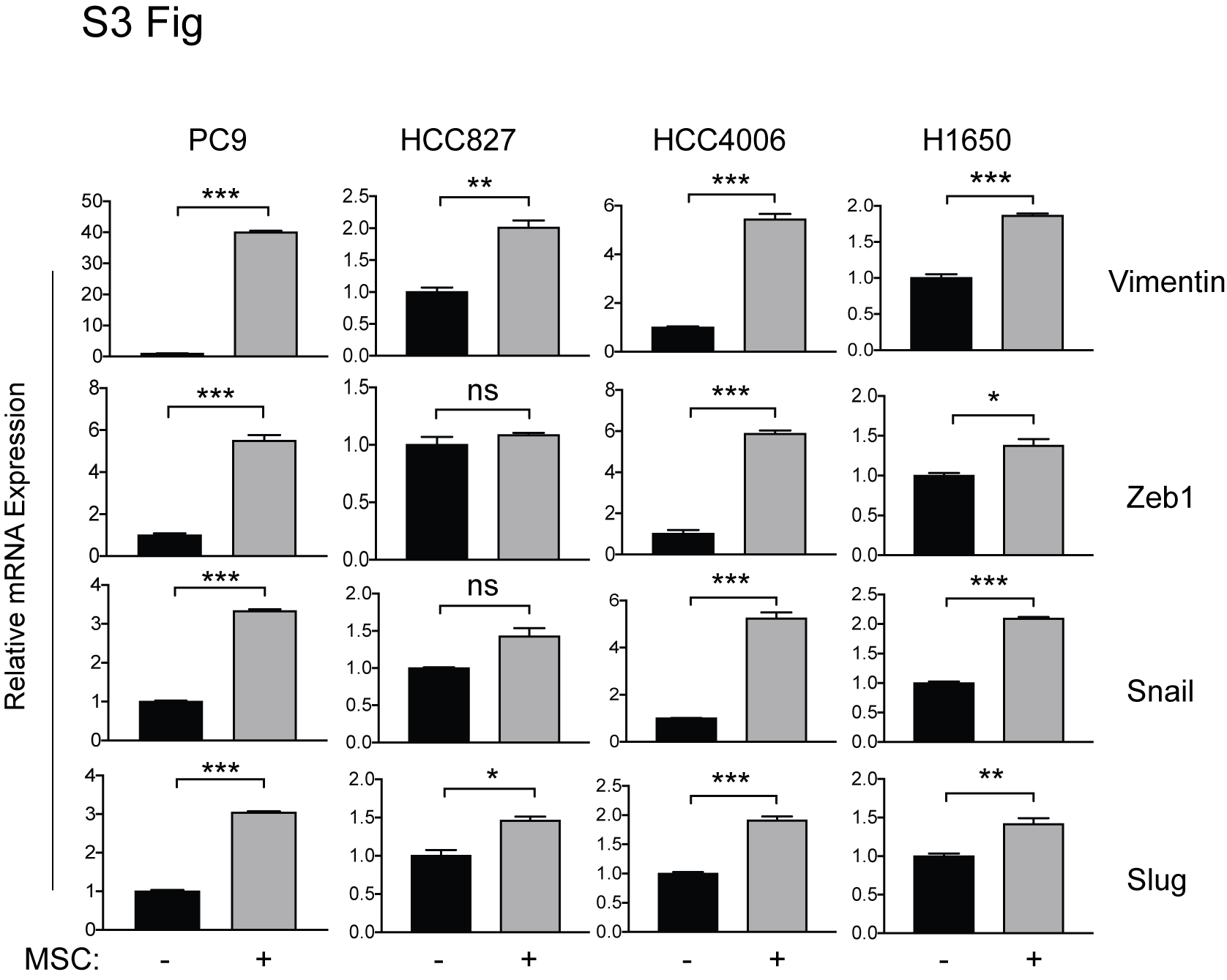

Supplement: S3 Fig — HCC827, HCC4006, H1650 and PC9 lung cancer cells were cultured with or without MSCs followed by FACS sorting. RT-PCR for indicated EMT markers was performed with lung cancer cells isolated from single culture compared to lung cancer cells sorted from co-culture with MSCs. Statistical analysis was performed using unpaired two-tail t test. *p<0.05, **p<0.01, ***p<0.001; ns = not significant. All assays were done in triplicate. (TIF) [file pone.0241423.s003.tif]

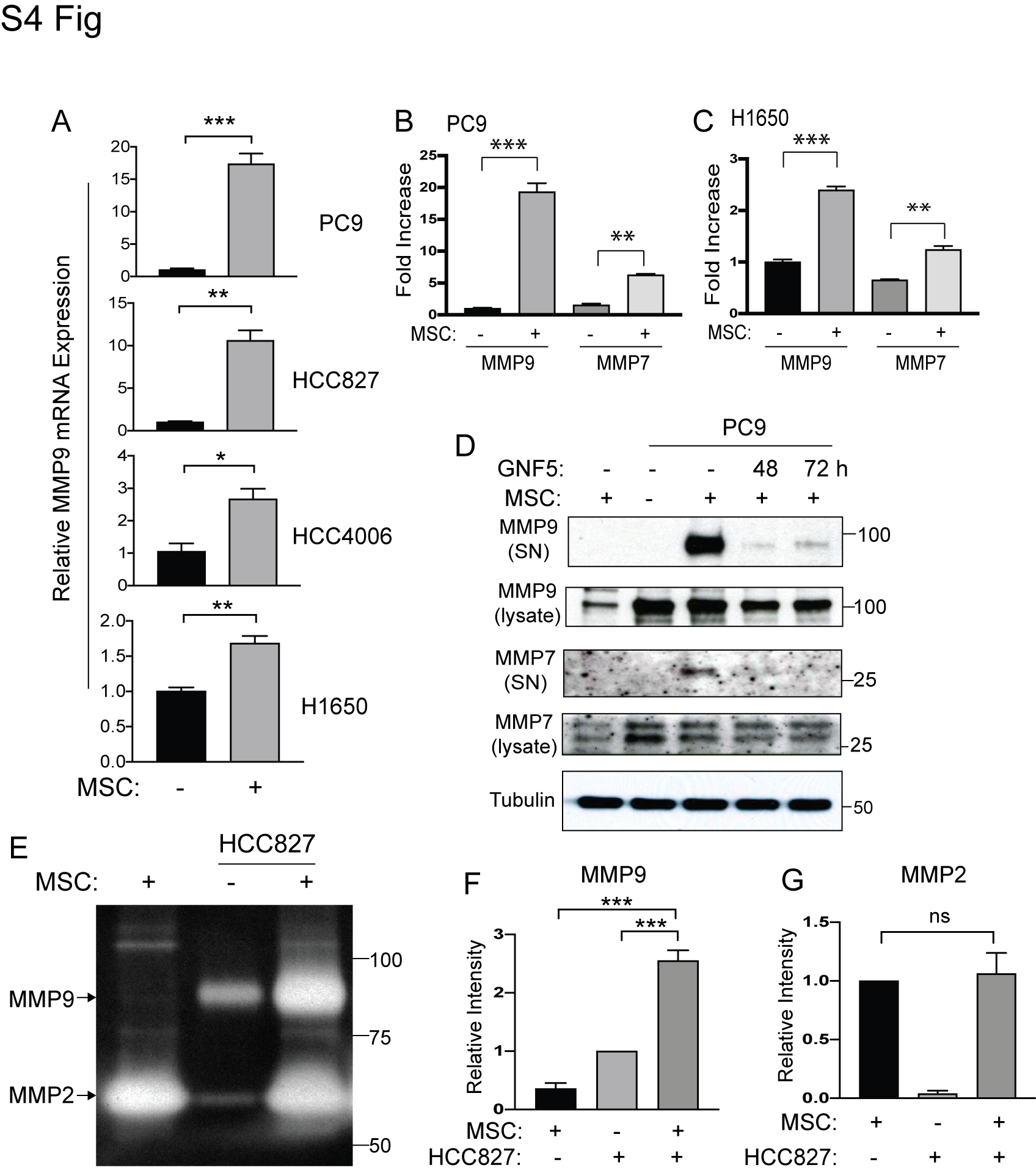

Supplement: S4 Fig — (A) RT-PCR for MMP9 mRNA expression in PC9, HCC827, HCC4006, and H1650 lung cancer cells cultured with or without MSCs followed by FACS sorting. Statistical analysis was performed using unpaired two-tail t test. *p<0.05, **p<0.01, ***p<0.001. All assays were done in triplicate. (B-C) RT-PCR for MMP9 and MMP7 mRNA expression in PC9 (B) and H1650 (C) cancer cells cultured with or without MSCs followed by FACS sorting. Statistical analysis was performed using One-way ANOVA followed by Tukey’s multiple comparison post hoc analysis (***p<0.001; **p<0.01). (D) PC9 cells were cultured with or without MSCs in the presence or absence of ABL kinase inhibitor GNF5 (5 μM) for 48 or 72h. Culture supernatants (SN) from MSC alone or PC9 co-cultured with or without MSC in the presence or absence of GNF5 were analyzed for MMP9 and MMP7 proteins. Total lysates were blotted with MMP9, MMP7 and tubulin. (E) Culture supernatants from MSCs, HCC827 single culture, or MSC+HCC827 co-culture were analyzed for MMP9 activity by gelatin-zymography assay. MMP9 and MMP2 gelatin digestion bands were indicated. (F-G) Quantification of MMP9 (F) and MMP2 (G) was carried out by Fiji software. Statistical analysis was performed using One-way ANOVA followed by Tukey’s multiple comparison post hoc testing. (***p<0.001; ns = not significant). Error bars represent ± SEM (n = 3). (TIF) [file pone.0241423.s004.tif]

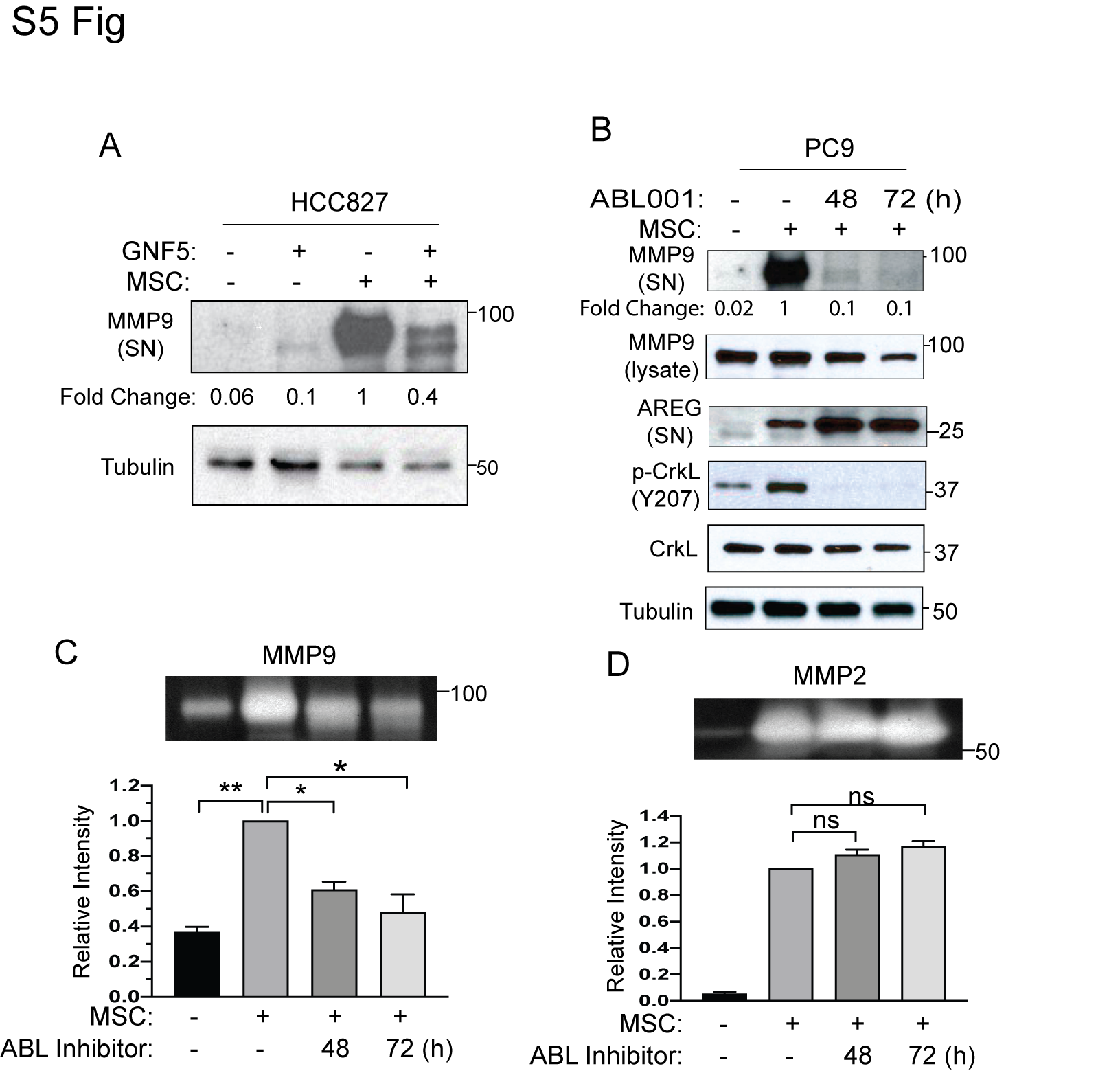

Supplement: S5 Fig — (A) HCC827 cells were cultured with or without MSCs and in the absence or presence of ABL allosteric inhibitor GNF5 (10 μM) for 72 h. Culture supernatants (SN) were analyzed for MMP9 protein and normalized to tubulin presented as fold change. (B) PC9 cells were cultured with or without MSCs and in the presence or absence of ABL allosteric inhibitor ABL001 (5 μM) for 48 and 72 h. Culture supernatants (SN) were analyzed for MMP9 and AREG proteins. MMP9 proteins in supernatant were normalized to MMP9 proteins in the lysate and presented as fold change. Total cell lysates were also analyzed with the indicated antibodies. (C-D) Culture supernatants collected from HCC827 cells cultured with or without MSCs in the presence or absence of ABL allosteric inhibitors ABL001 were analyzed for MMP9 activity on gelatin zymography. A representative zymographic band is shown (top), and quantifications of corresponding bands (bottom) was done by Fiji software. Statistical analysis was performed using One-way ANOVA followed by Tukey’s multiple comparison post hoc testing (**p<0.01, *p<0.05, ns = not significant). Error bars represent ± SEM (n = 2). (TIF) [file pone.0241423.s005.tif]

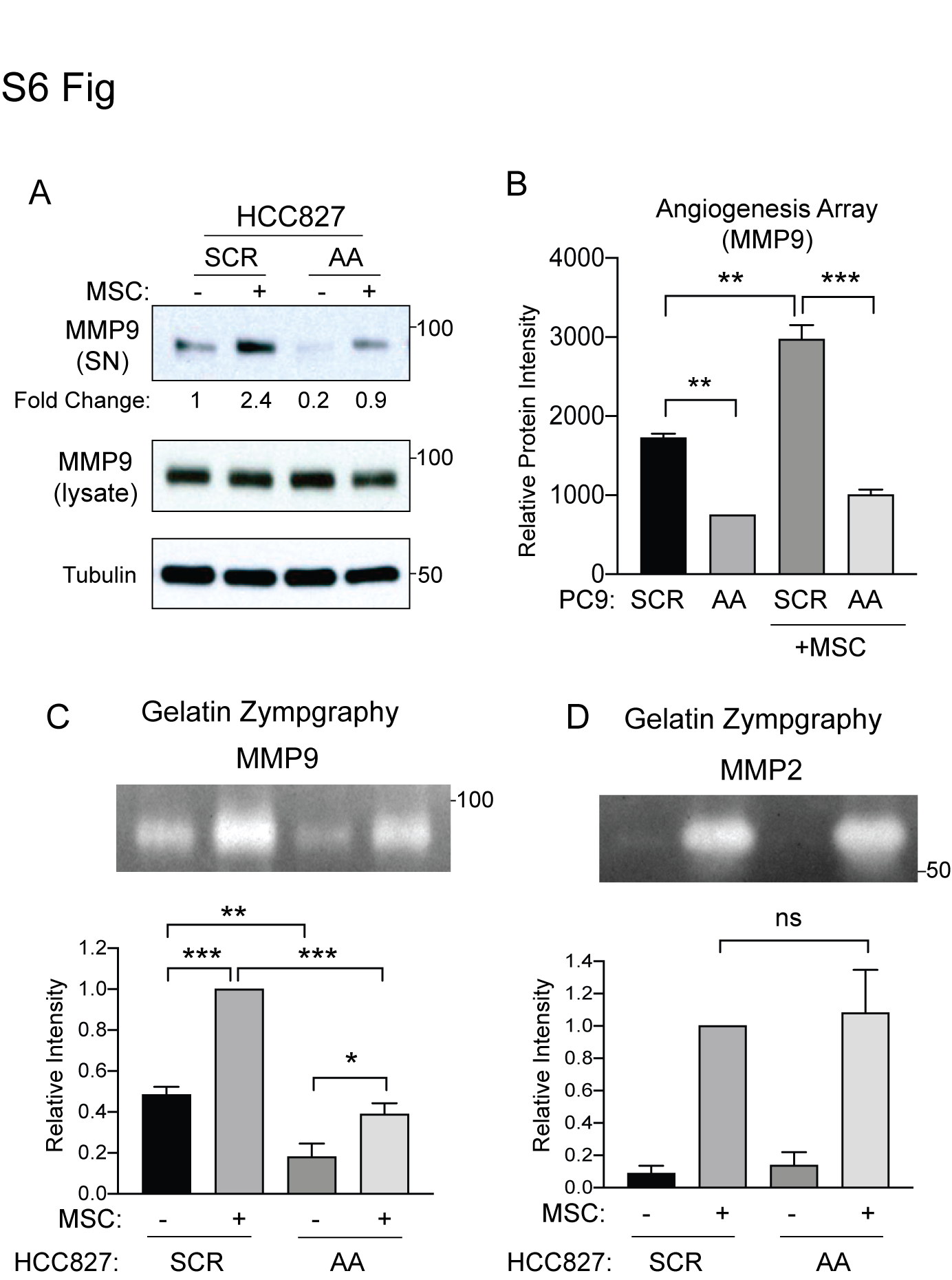

Supplement: S6 Fig — (A) HCC827 lung cancer cells were transduced with either scramble control shRNA (SCR) or shRNAs specific for ABL1 and ABL2 (AA). Cells were then cultured with or without MSCs. Culture supernatants (SN) were analyzed for MMP9 protein and normalized to MMP9 in lysates and expressed as fold change. (B) PC9-SCR and PC9-AA cells were cultured with or without MSCs, and culture supernatants were analyzed for MMP9 protein using the Angiogenesis Array performed in duplicates. Statistical analysis was performed using One-way ANOVA followed by Tukey’s multiple comparison post hoc testing (**p<0.01; ***p<0.001). (C-D) Cell culture supernatants from HCC827 cells transduced with shRNA control (SCR) or shRNAs-specific against ABL1+ABL2 (AA) were cultured with or without MSCs and were then analyzed for MMP9 (C) and MMP2 (D) enzymatic activity using gelatin-zymography. Representative zymographic band are shown (top), and quantification of corresponding bands (bottom) was carried out by Fiji software. Statistical analysis was performed using One-way ANOVA followed by Tukey’s multiple comparison post hoc testing (***p<0.001, **p<0.01, ns = not significant). Error bars represent ± SEM (n = 2). (TIF) [file pone.0241423.s006.tif]
