# Supplementary material for: Mesenchymal stem cells promote metastasis through activation of an ABL-MMP9 signaling axis in lung cancer cells
Source: PLoS One. 2020 Oct 29;15(10):e0241423. doi: 10.1371/journal.pone.0241423 (PMC7595271; doi:10.1371/journal.pone.0241423)

A note for preparing original gels:

Western blotting membranes corresponding to an appropriate range of protein size were used for each specific antibody.

All films were scanned with a resolution of 300 ppi and visualized with Adobe Photoshop under “Auto tone” and “Auto contrast” setting. Occasionally slightly adjustment to Brightness were applied to the entire membrane.

Uncropped original membranes are prepared including the whole height and width of the membrane used for that specific antibody. An “X” indicates lanes that are not relevant nor included in the figures. Relevant bands were labeled same as in the figures. An arrow indicates the appropriate protein size for that particular protein.

Original Gel-1

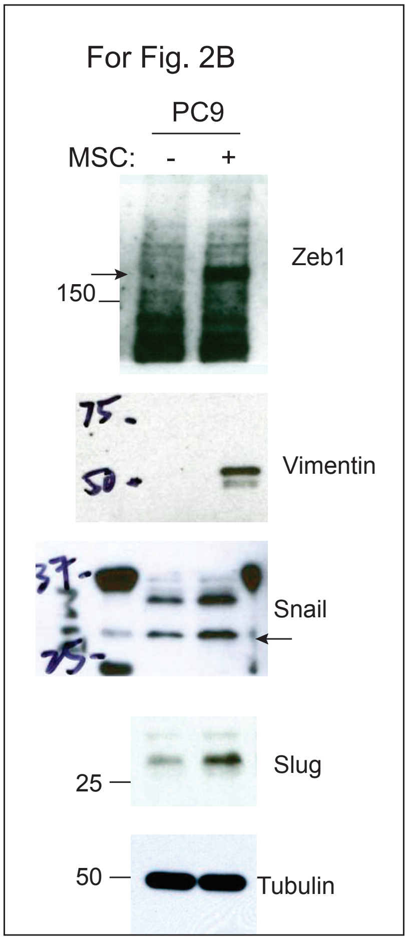

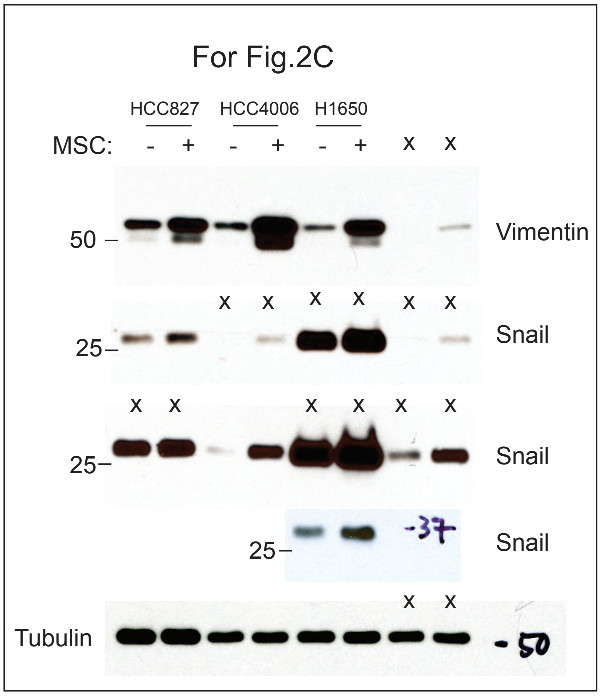

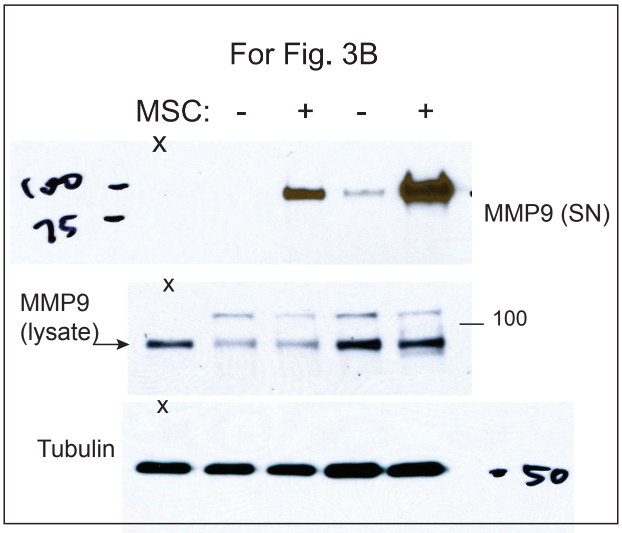

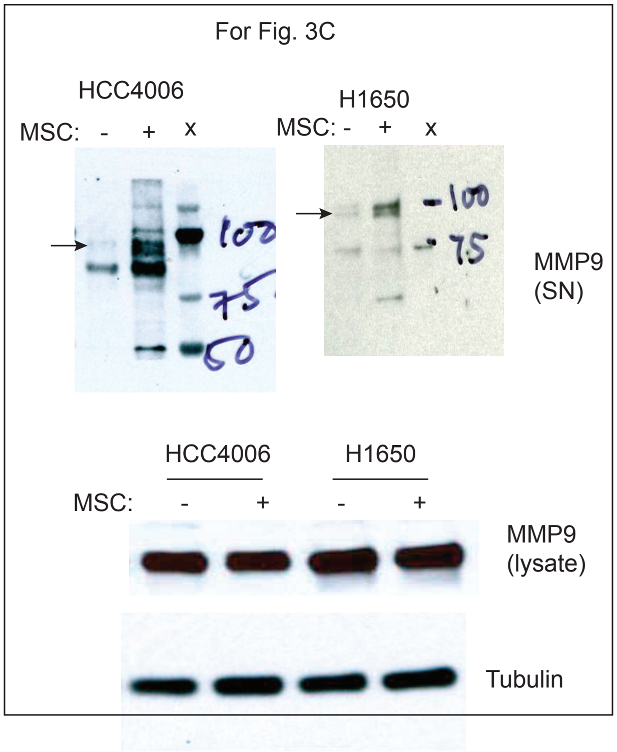

Original Gel-2

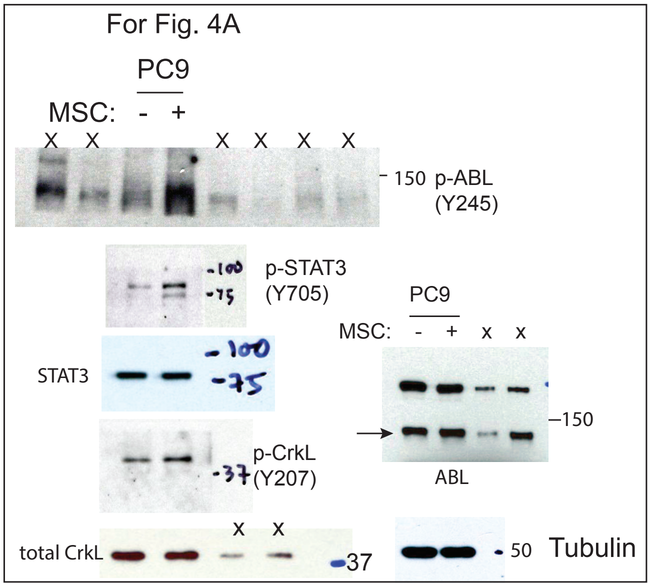

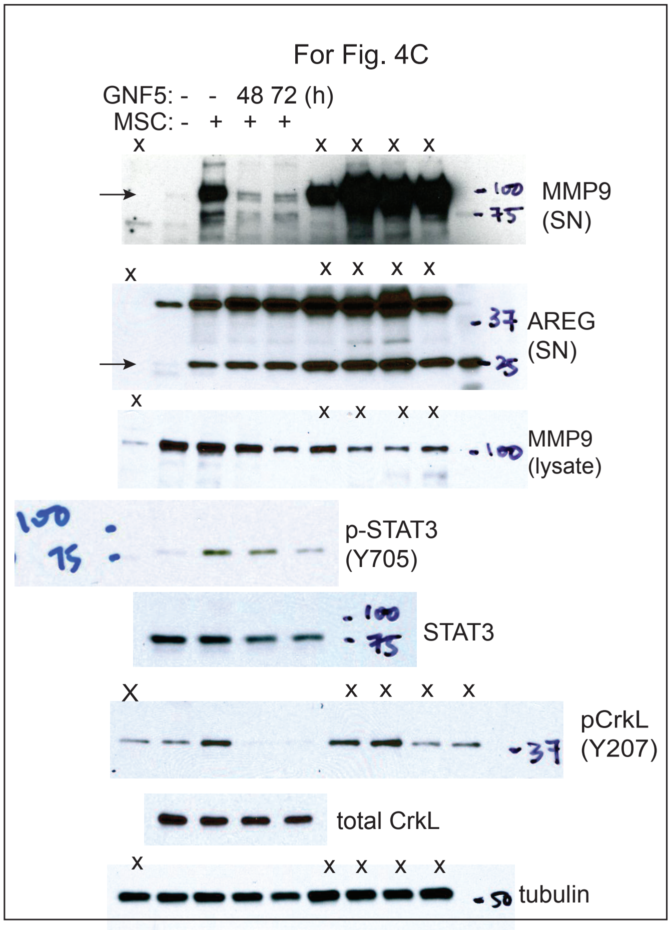

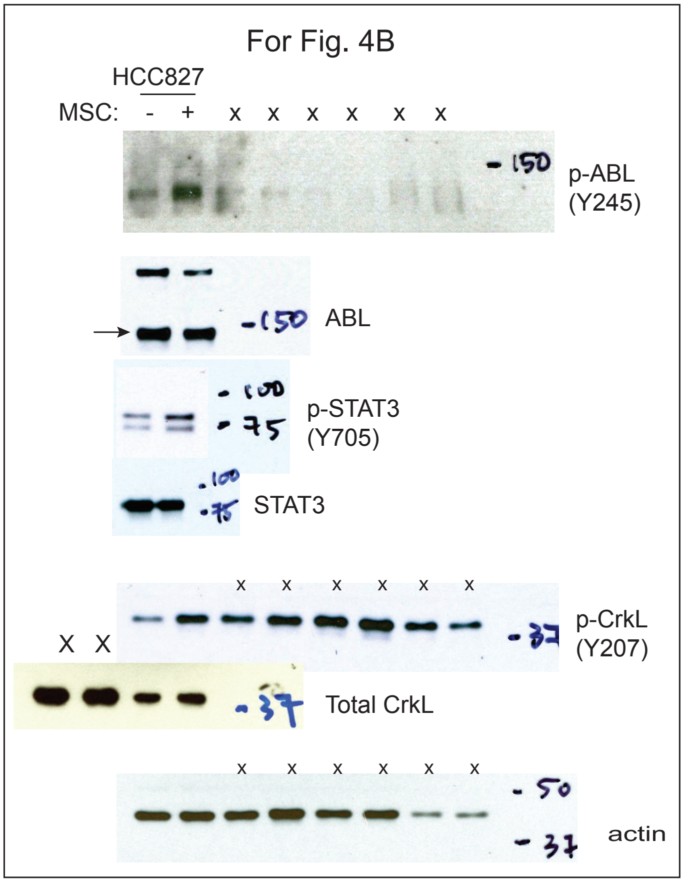

Original Gel-3

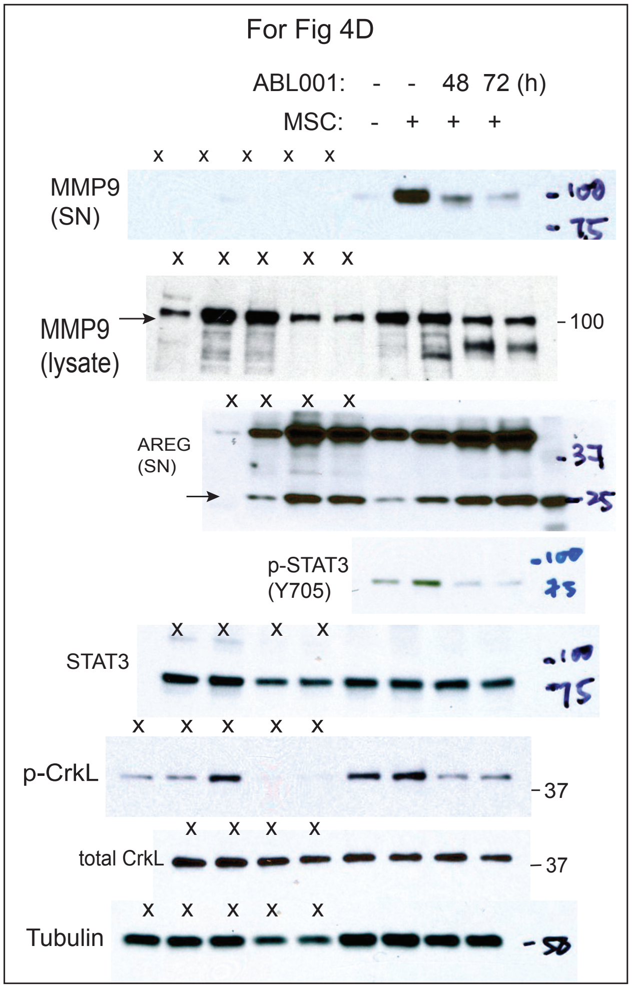

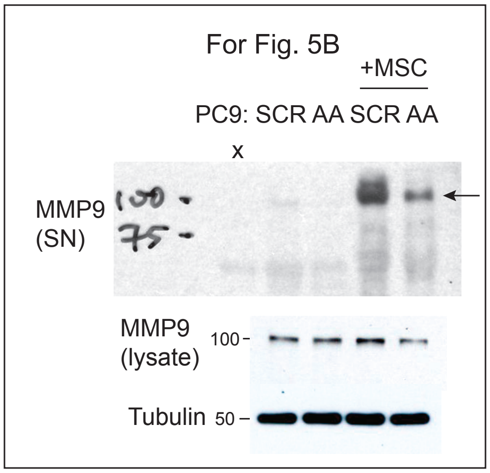

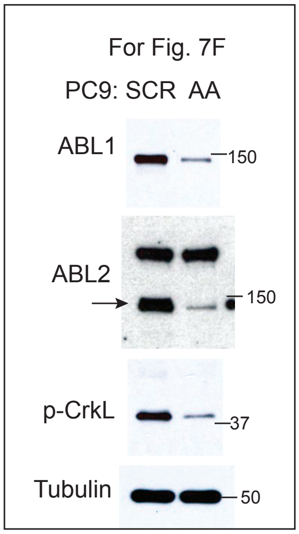

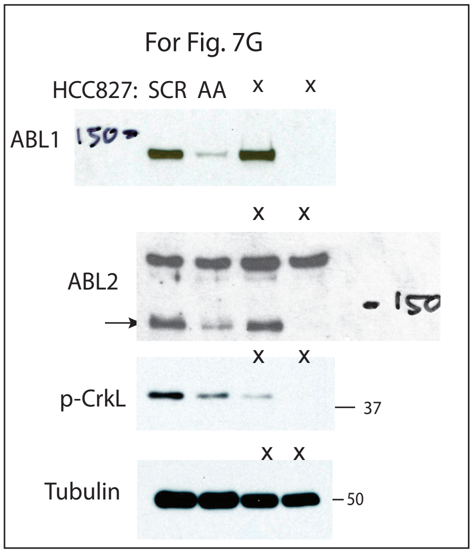

Original Gel-4

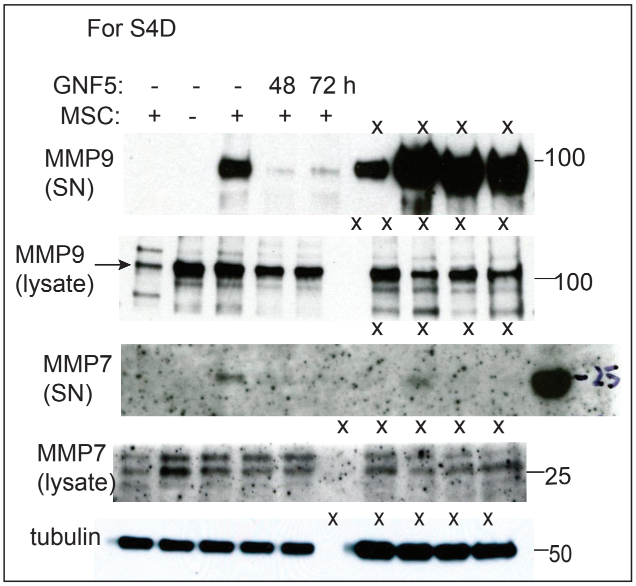

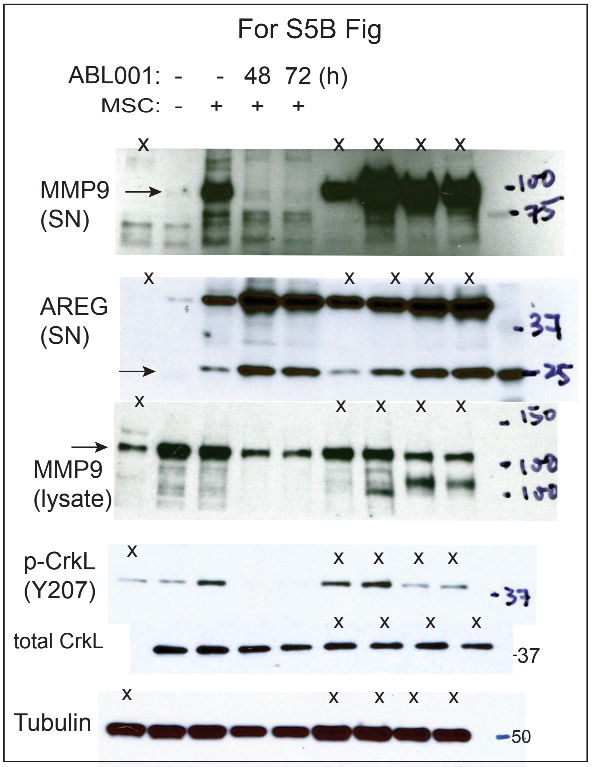

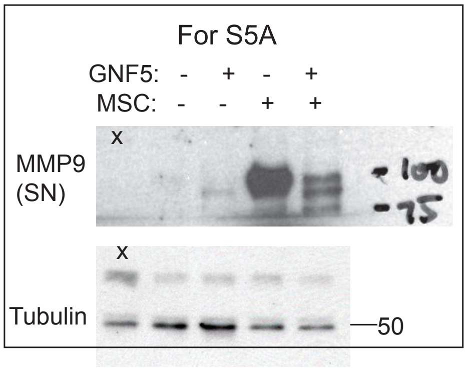

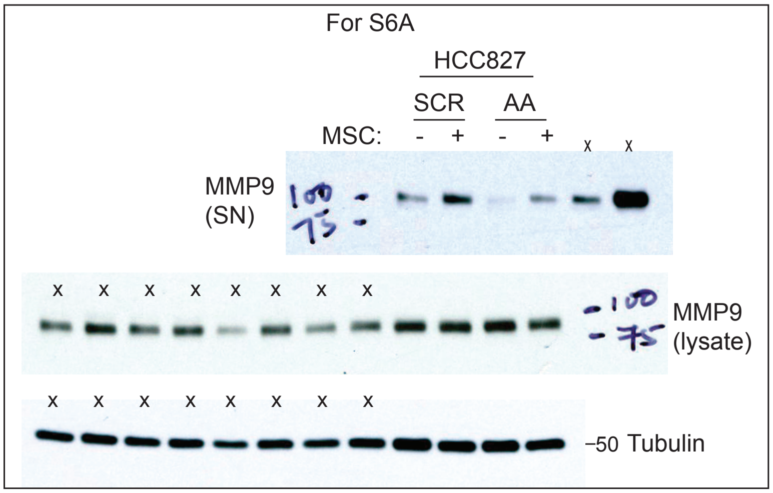

Original Gel-5

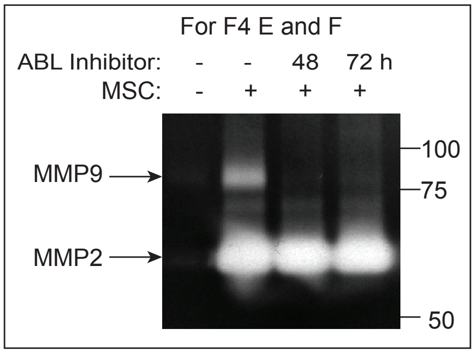

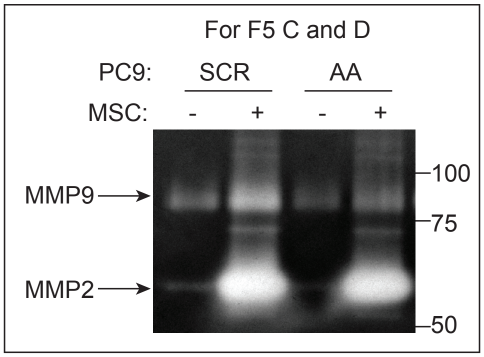

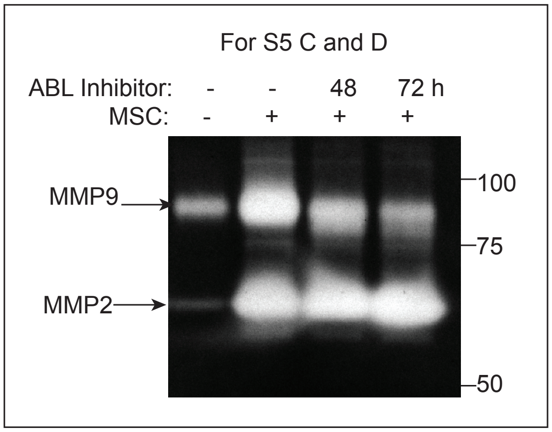

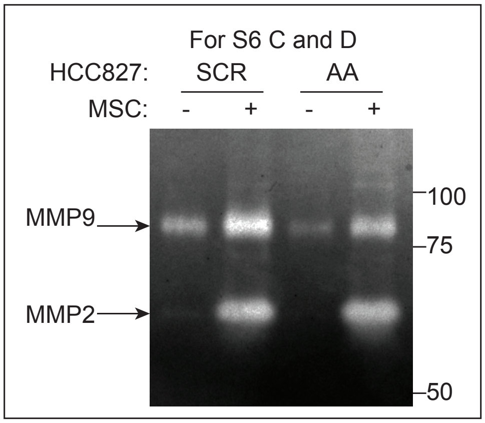

Supplement: S1 File — (PDF) [file pone.0241423.s009.pdf]
