# Supplementary material for: Mesenchymal stem cells promote metastasis through activation of an ABL-MMP9 signaling axis in lung cancer cells
Source: PLoS One. 2020 Oct 29;15(10):e0241423. doi: 10.1371/journal.pone.0241423 (PMC7595271; doi:10.1371/journal.pone.0241423)
Supplement: Attachment — Submitted filename: Response to Reviewers.pdf [file pone.0241423.s010.pdf]

Response to Reviewers and Editor (# PONE-D-20-19649)

We thank the Editor and Reviewers for their valuable feedback on our manuscript entitled "Mesenchymal stem cells promote metastasis through activation of an ABL-MMP9 signaling axis in lung cancer cells". We are gratified that the reviewers are enthusiastic about the study. We have generated new data to address the questions and minor modifications raised by the reviewers. The new data included in the revised manuscript strengthen the manuscript's conclusions. The point-by-point responses to the reviewers' comments are addressed below.

Academic Editor

Please ensure that your manuscript meets PLOS ONE's style requirements, including those for file naming.

RESPONSE: We have followed the journal guard line to meet the PLOS ONE's style requirements.

Please provide additional information about each of the cell lines used in this work, including any quality control testing procedures (authentication, characterisation, and mycoplasma testing).

RESPONSE: Most of the cell lines used were purchased from ATCC. The PC9 lung adenocarcinoma cell line was obtained from Dr. Joan Massague, and was provided by the MACF/Memorial Sloan-Kettering Institute facility. Cell lines were subjected to short tandem repeat (STR) profiling through the Duke University DNA Analysis Facility Human cell line authentication (CLA) service to confirm their authenticity. Upon receipt, cell lines were grown and multiple vials were frozen after the first passage of the initial vial to ensure that only early passages were used for all experiments. New vials were employed monthly to reconstitute the working population. Cell lines were routinely checked for mycoplasma and only mycoplasma-free cell lines were used. Fetal Bovine Serum (FBS) employed in the cell culture media are extensively tested prior to purchasing the lots to ensure that the serum is endotoxin-free. This information has been added to the Materials and Methods section.

Please provide additional information about the HEK293T cells used in this work, including the source any quality control testing procedures (authentication, characterisation, and mycoplasma testing).

RESPONSE: The HEK293T cells were obtained from ATCC and provided by the Duke Cell Culture facility where the cells were tested for mycoplasma, authenticated and characterized as detailed above. This information has been added to the "Materials and Methods" section.

To comply with PLOS ONE submissions requirements, please provide the method of euthanasia in the Methods section of your manuscript.

RESPONSE: Following Duke Institutional Animal Care and Use Committee humane euthanasia rule, we euthanized mice with CO_2 followed by secondary physical disruption (exsanguination). This information has been added to the Materials and Methods section.

PLOS ONE now requires that authors provide the original uncropped and unadjusted images underlying all blot or gel results reported in a submission's figures or Supporting Information files.

RESPONSE: We have provided all the original gel images in a single PDF file that is included in the Supporting Information. All figures adhere fully to the guidelines.

Reviewer #1

We thank the reviewer for stating that the "*study and experiments have been well designed and are overall presented clearly and effectively*". We have addressed the few minor comments and recommendations (*italics*) raised, and which have been added to the revised manuscript.

MMP7 transcript expression was also significantly increased following MSC/NSCLC co-culture (Figure 3A). The authors should clarify why only MMP9 was pursued.

RESPONSE: Whereas MMP9 expression was significantly increased in the PC9 lung cancer cells compared to MSCs following MSC/NSCLC co-culture, increased MMP7 expression following co-culture was predominantly detected in MSC cells and to a much lesser extent in the PC9 lung cancer cells. We further confirmed the findings in Figure 3A by directly comparing expression of MMP7 to MMP9 after co-culture following FACS sorting of PC9 and H1650 lung cancer cells and analysis of mRNA and protein expression as shown in Supplemental Figures 4B-C. Thus, we focused on MMP9 because this protease was consistently increased in the cancer cells following MSC/NSCLC co-culture.

In Figure 4A and B, pCrkL and pSTAT3 are used as readouts for ABL kinase activity, but only pCrkL is presented in Figure 4C and D to validate the effect of treatment with GNF5 or ABL001. Levels of pSTAT3 should be added to these panels for consistency if possible.

RESPONSE: We have performed p-Stat3, total Stat3 and total CrkL western blot analysis as requested and have added the new data to Figure 4C and 4D.

Line 297 refers to "Affimetrix." Please check whether this should be "Affymetrix."

RESPONSE: We have corrected Affimetrix to Affymetrix.

In contrast to the PC9 cells (Figure 7C), the impact of ABL depletion on survival in HCC827 is not mentioned. Even if not significant, the data for HCC827 could be included for completion, and the authors could discuss potential reasons for the difference.

RESPONSE: HCC827 cells are larger in size than PC9 cells and aggregate in clusters that can elicit vessel obstruction and rapid death in mice injected with the same injection protocol as that used for PC9 cell injection. Therefore, we injected a reduced number of HCC827 cells ($1x10^5$) by intracardiac route to avoid blood vessel clotting that often led to death of mice. Furthermore, the doubling rate of HCC827 cells is significantly slower than PC9 cells. Therefore, injection of $1x10^5$ HCC827 cells in mice would necessitate a much longer timeline for the mouse survival studies which is estimated would be 9 to 12 months. Given the excessive extended timeline we were not able to carry out this experiment with HCC827 cells.

Please specify that the bone marrow MSCs from Lonza are human origin in the Materials and Methods.

RESPONSE: The bone marrow derived MSCs are human origin. We have added this information to the "Materials and Methods" section.

Reviewer #2

We thank the reviewer for stating that the manuscript "*is a very interesting report of ABL kinase modulating EGFR-mutant non-small cell lung cancer (NSCLC) invasiveness and metastatic colonization through metalloproteinase (MMP) upregulation*", and that the "*work is well designed, well performed and I believe it will appeal to PlosOne audience*". We have addressed the minor suggestions below:

1 – *BM-MSC tropism to inflammatory and cancer sites is well reported. However, the exactly extent that it could happen in pathophysiological conditions it is not known. The work shows a correlation of high MMP9 expression, which is upregulated by stromal BM-MSC, and poor patient outcome. Have authors assessed how many of these patients have significant tumor stroma associated-lung adenocarcinoma?*

RESPONSE: We thank the author for this comment and agree that it would be of interest to more fully elucidate how tumor stroma infiltration correlates with patient outcomes in lung adenocarcinoma.

Unfortunately, the patient survival data showing a correlation of high MMP9 mRNA expression with poor survival outcomes is not amenable for such interrogation. As described in the methods section, these data were analyzed using the KMPlot analysis tool originally developed by Győrffy et al, which houses a collection of patient datasets linking tumor gene expression (microarray) with survival outcomes (1). As the microarray data used in these datasets does not provide corresponding information on stromal involvement (for example, through IHC or H&E staining/imaging), we are unable to determine from this particular dataset if patients with high tumor MMP9 expression present with increased stromal infiltration.

A few recent reports have sought to explore the relationship between tumor vs stroma (primarily through H&E tissue staining/analysis) in the context of patient survival. For example, Costas et al used immunohistochemistry to analyze stromal involvement in a cohort of NSCLC patients with available survival data and found that the presence of fibrotic stroma correlated with increased mortality (2). Zhang and colleagues, using a similar approach in a larger cohort of 404 NSCLC patients, defined a measure termed the "tumor-stroma ratio" (TSR) and found that stroma-high tumors were associated with an increased risk of relapse following surgical resection and poor prognosis compared to patient tumors with minimal stromal infiltration (3). These results are further supported by a more recent study of TSR in NSCLC patients, which came to similar conclusions (4). While these human patient data do not directly examine tumor MMP9 expression in the context of stromal recruitment to tumors, at least one study identified a correlative relationship between tumor MMP9 expression and increased stromal involvement in NSCLC (5). As most of these studies are largely correlative, we agree with the reviewer that future studies in this area which are beyond the scope of this work, should be undertaken to evaluate a causal relationship between patient survival and stromal involvement. Moreover, in order to ascertain which cell type is responsible for increased MMP9 expression in patient specimens, it would be necessary to use single cell RNA-seq as current databases do not indicate whether increased MMP9 expression is associated with lung tumor cells, MSCs, immune cells, or other cell types in the tumor microenvironment.

2 - *It is known that cell passages could influence BM-MSC phenotype and cell outcome. Could the authors cite in which passage cells were used at the material and methods?*

RESPONSE: We used BM-MSC cells at passage 5 or 6. We have added this information to the "Materials and Method" section.

3 - *In many protein blots, it is shown phosphorylated protein isoform but not total protein isoform. It is known that alterations within the total amount of protein available could affect the interpretation of the measurement of protein phosphorylation status. In line with this, the normalization of a phosphorylated protein to its total expression allows the ratio of phosphorylated proteins to be assessed. Do the authors believe that this normalization is not necessary?*

RESPONSE: We agree with the reviewer that normalization of a phosphorylated protein to its total expression allows the ratio of phosphorylated proteins to be assessed, and therefore we have performed the Western blots for total ABL, STAT3 and CRKL proteins (included in Figure 4). We have normalized all the phospho-proteins to their corresponding total proteins. Also, we have re-quantified the supernatant MMP9 protein to their total lysate MMP9 level, and have included these blots in the revised figures.

4 - *It is very important to watch for figure definition. Some legends are too small or without resolution. Mainly graphic axis legends. It must be improved before final version.*

RESPONSE: We have improved the figure definition and enhanced the legend size. We created the figures using Adobe Illustrator, and enhanced the graphic labeling. These changes have improved the clarity and definition of the figures.

5 - *Data regarding lung cancer survival rate in lines 56-57 should be referenced.*

RESPONSE: We have included several recent review references on lung cancer survival rates as requested, which indicate that the 5-year survival rate for lung cancer is estimated at less than 20% (7, 8).

6. PLOS authors have the option to publish the peer review history of their article, this will include your full peer review and any attached files.

RESPONSE: Yes, you may publish the review history.

References:

1. Gyorffy B, Surowiak P, Budczies J, Lanczky A. Online survival analysis software to assess the prognostic value of biomarkers using transcriptomic data in non-small-cell lung cancer. PLoS One. 2013;8(12):e82241.
2. Costas, L. M., Alcaraz, J., Benchea, I., Carrasco, J. L., de Cos, J. S., Castro, M. A. G., ... & Sauleda, J. (2017). Effects of tumor stroma and inflammation on survival of stage I-IIp lung cancer.
3. Zhang, T., Xu, J., Shen, H., Dong, W., Ni, Y., & Du, J. (2015). Tumor-stroma ratio is an independent predictor for survival in NSCLC. International journal of clinical and experimental pathology, 8(9), 11348.
4. Xi, K. X., Wen, Y. S., Zhu, C. M., Yu, X. Y., Qin, R. Q., Zhang, X. W., ... & Zhang, L. J. (2017). Tumor-stroma ratio (TSR) in non-small cell lung cancer (NSCLC) patients after lung resection is a prognostic factor for survival. Journal of thoracic disease, 9(10), 4017.
5. Minamoto, H., Antonangelo, L., Da Silva, A. G. P., Gallo, C. P., De Andrade e Silva, F. B., Fenezelian, S., ... & Capelozzi, V. L. (2003). Tumour cell and stromal features in metastatic and non-metastatic non-small cell lung carcinomas. Histopathology, 43(5), 427-443.
6. Cheng B, Xiong S, Li C, Liang H, Zhao Y, Li J, et al. An annual review of the remarkable advances in lung cancer clinical research in 2019. J Thorac Dis. 2020;12(3):1056-69.
7. Lin HT, Liu FC, Wu CY, Kuo CF, Lan WC, Yu HP. Epidemiology and Survival Outcomes of Lung Cancer: A Population-Based Study. Biomed Res Int. 2019;2019:8148156.
